# Expression and Prognostic Significance of CD47–SIRPA Macrophage Checkpoint Molecules in Colorectal Cancer

**DOI:** 10.3390/ijms22052690

**Published:** 2021-03-07

**Authors:** Akane Sugimura-Nagata, Akira Koshino, Satoshi Inoue, Aya Matsuo-Nagano, Masayuki Komura, Miho Riku, Hideaki Ito, Akihito Inoko, Hideki Murakami, Masahide Ebi, Naotaka Ogasawara, Toyonori Tsuzuki, Satoru Takahashi, Kunio Kasugai, Kenji Kasai, Shingo Inaguma

**Affiliations:** 1 Division of Gastroenterology, Department of Internal Medicine, Aichi Medical University School of Medicine, Nagakute 480-1195, Japan; nagata.akane.240@mail.aichi-med-u.ac.jp (A.S.-N.).; koshino.akira.255@mail.aichi-med-u.ac.jp (A.K.); inoue.satoshi.002@mail.aichi-med-u.ac.jp (S.I.); ebi.masahide.814@mail.aichi-med-u.ac.jp (M.E.); ogasawara.naotaka.667@mail.aichi-med-u.ac.jp (N.O.); kasugai.kunio.527@mail.aichi-med-u.ac.jp (K.K.); 2 Department of Experimental Pathology and Tumor Biology, Nagoya City University Graduate School of Medical Sciences, Nagoya 467-8601, Japan; aya.ngn@med.nagoya-cu.ac.jp (A.M.-N.); komura@med.nagoya-cu.ac.jp (M.K.); sattak@med.nagoya-cu.ac.jp (S.T.); 3 Department of Pathology, Aichi Medical University School of Medicine, Nagakute 480-1195, Japan; mriku@aichi-med-u.ac.jp (M.R.); itou.hideaki.820@mail.aichi-med-u.ac.jp (H.I.); inoko.akihito.288@mail.aichi-med-u.ac.jp (A.I.); murakami.hideki.542@mail.aichi-med-u.ac.jp (H.M.); kkasai@aichi-med-u.ac.jp (K.K.); 4 Surgical Pathology, Aichi Medical University School of Medicine, Nagakute 480-1195, Japan; tsuzuki@aichi-med-u.ac.jp; 5 Department of Pathology, Nagoya City East Medical Center, Nagoya 464-8547, Japan; 6 Educational Research Center for Advanced Medicine, Nagoya City University Graduate School of Medical Sciences, Nagoya 467-8601, Japan

**Keywords:** colorectal cancer (CRC), immunohistochemistry, macrophage checkpoint, CD47, signal regulatory protein-alpha (SIRPA)

## Abstract

Despite the confirmed anti-cancer effects of T-cell immune checkpoint inhibitors, in colorectal cancer (CRC) they are only effective in a small subset of patients with microsatellite-unstable tumors. Thus, therapeutics targeting other types of CRCs or tumors refractory to T-cell checkpoint inhibitors are desired. The binding of aberrantly expressed CD47 on tumor cells to signal regulatory protein-alpha (SIRPA) on macrophages allows tumor cells to evade immune destruction. Based on these observations, drugs targeting the macrophage checkpoint have been developed with the expectation of anti-cancer effects against T-cell immune checkpoint inhibitor-refractory tumors. In the present study, 269 primary CRCs were evaluated immunohistochemically for CD47, SIRPA, CD68, and CD163 expression to assess their predictive utility and the applicability of CD47–SIRPA axis-modulating drugs. Thirty-five percent of the lesions (95/269) displayed CD47 expression on the cytomembrane of CRC cells. CRCs contained various numbers of tumor-associated immune cells (TAIs) with SIRPA, CD68, or CD163 expression. The log-rank test revealed that patients with CD47-positive CRCs had significantly worse survival than CD47-negative patients. Multivariate Cox hazards regression analysis identified tubular-forming histology (hazard ratio (R) = 0.23), age < 70 years (HR = 0.48), and high SIRPA-positive TAI counts (HR = 0.55) as potential favorable factors. High tumor CD47 expression (HR = 1.75), lymph node metastasis (HR = 2.26), and peritoneal metastasis (HR = 5.80) were cited as potential independent risk factors. Based on our observations, CD47–SIRPA pathway-modulating therapies may be effective in patients with CRC.

## 1. Introduction

The discovery of T-cell immune checkpoint inhibitors launched a new era in cancer therapy. Moreover, evidence supporting the anti-cancer effects of these drugs is accumulating [1,2]. However, in colorectal cancer (CRC), the application of these therapies is currently limited to a small subset of patients with microsatellite-unstable tumors [3].

Macrophages are effector cells of the innate immune system that phagocytose bacteria and secrete mediators for pro-inflammatory and anti-microbial effects. Macrophages eliminate diseased or damaged cells through programmed cell death and serve as antigen-presenting cells. Therefore, macrophage checkpoints including CD47–signal regulatory protein-alpha (SIRPA) signaling are considered to play important roles in cancer surveillance [4,5].

CD47 is a heavily glycosylated, ubiquitously expressed cell surface protein in the immunoglobulin superfamily. Its molecular structure includes an extracellular immunoglobulin variable-like domain, a transmembrane spanning domain, and a short, alternatively spliced cytoplasmic tail [6]. Although CD47 was first identified as a membrane protein involved in β3 integrin-mediated signaling on leukocytes [7], it is reported to interact with SIRPA, thrombospondin-1, and other molecules to regulate various cellular functions including cell migration, axon extension, cytokine production, and T-cell activation [8,9,10,11,12]. In a variety of hematologic and solid malignancies, aberrant expression of CD47 was reported to independently correlate with poor clinical outcomes [13,14].

SIRPA is a member of the signal-regulatory-protein family, and it was first identified as a membrane protein present mainly on macrophages and myeloid cells that is associated with the Src homology region 2 domain-containing phosphatases SHP-1 and SHP-2 [15]. SIRPA contains three immunoglobulin-like domains, a single transmembrane region, and a cytoplasmic region containing four Tyr residues within immunoreceptor tyrosine-based inhibitory motifs [15]. CD47 has been identified as a ligand for SIRPA. The binding of CD47 to SIRPA on macrophages and dendritic cells results in the inhibition of phagocytosis. Thus, CD47 provides a potent “don’t eat me” signal that allows tumor cells to evade immune destruction by first-responder phagocytic cells and functions as a dominant macrophage checkpoint.

Agents that inhibit CD47–SIRPA signaling can induce the phagocytosis of cancer cells by macrophages, resulting in growth inhibition and regression in xenograft models [14,16,17]. Based on these observations, targeting CD47 is considered a novel immunotherapeutic strategy for several human cancers that are refractory to T-cell immune checkpoint inhibitors [18,19,20].

The present study examined the expression status of CD47 in CRCs. In addition, the expression of SIRPA and macrophage markers (CD68 and CD163) in tumor-associated immune cells (TAIs) was analyzed. The association of the expression of these proteins with clinicopathological features and clinical outcomes were analyzed to assess their potential for clinical use.

## 2. Results

### 2.1. Expression of CD47, SIRPA, CD68, and CD163 in Non-Neoplastic Colonic Mucosae and CRCs

**Figure 1:**
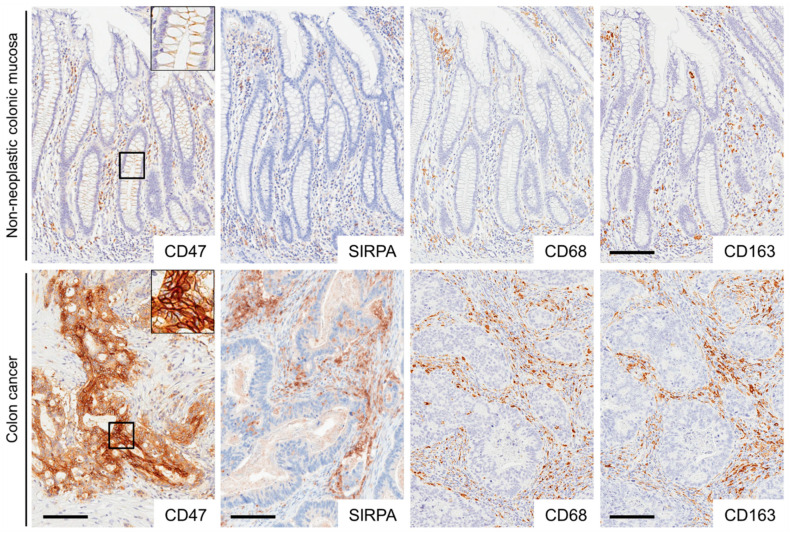
Representative images of CD47, SIRPA, CD68, and CD163 immunostaining in non-neoplastic colonic mucosae and colorectal cancer (CRC) tissues. CD47 was weakly expressed on the cytomembrane of non-neoplastic colonic epithelial cells. CRC cells expressed CD47 on the cytomembrane. Non-neoplastic colonic mucosae and colon cancer stroma contained immune cells positive for signal regulatory protein-alpha (SIRPA), CD68, or CD163. Bar, 100 μm.

Representative images for immunohistochemistry are presented in Figure 1 and Supplementary Materials, Figure S1. In non-neoplastic colonic mucosae, CD47 was weakly expressed on the cytomembrane of colonic epithelial cells. In addition, CD47-positive inflammatory cells were observed in the stroma. In total, 35% of the CRCs (95/269) exhibited CD47 expression on the cytomembrane of tumor cells. In non-neoplastic colonic mucosae as well as colon cancer stroma, TAIs positive for SIRPA, CD68, or CD163 were variably observed.

**Figure 2:**
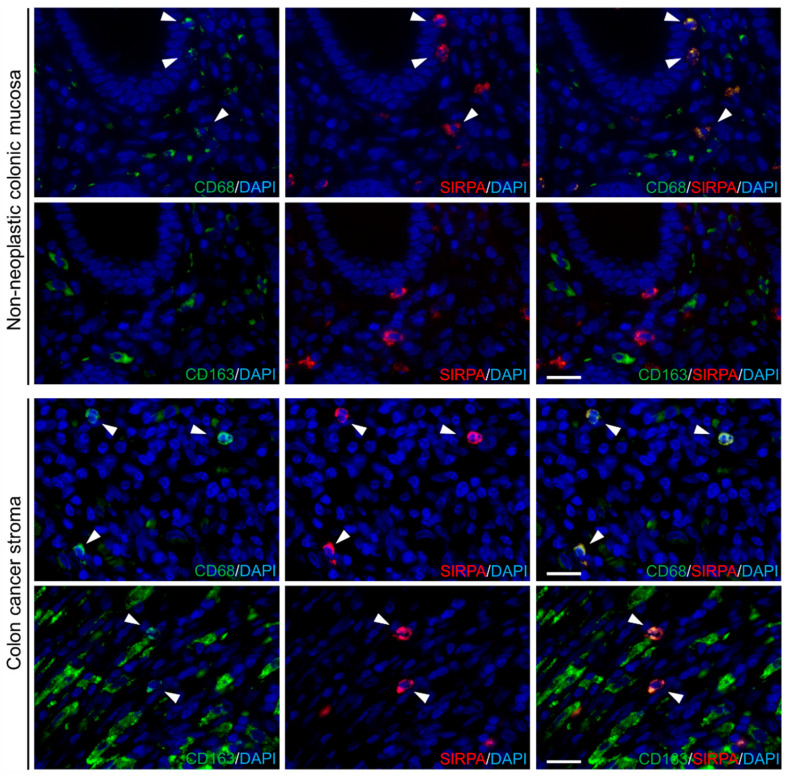
SIRPA was expressed in the subpopulation of CD68- and CD163-positive cells. SIRPA was expressed in the subset of CD68-positive cells in non-neoplastic colonic mucosae (upper panels). In colon cancer stroma, SIRPA expression was observed in both CD68- and CD163-positive tumor-associated immune cells (TAIs) (lower panels). Bar, 20 μm.

Representative images for fluorescent immunohistochemistry are presented in Figure 2. In non-neoplastic colonic mucosae, SIRPA was expressed in a subset of CD68-positive cells. In contrast, SIRPA was expressed in a subset of both CD68- and CD163-positive TAIs in CRC stroma.

**Figure 3:**
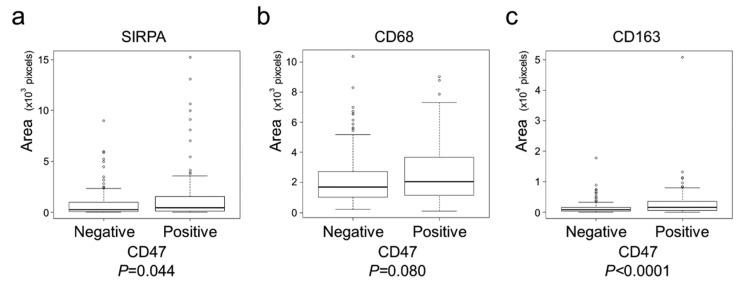
CD47-positive tumors contained significantly more SIRPA- or CD163-positive TAIs. (**a**) CD47-positive tumors contained significantly more SIRPA-positive TAIs (*p* = 0.044). (**b**) CD47-positive tumors tended to contain more CD68-positive TAIs (*p* = 0.080). (**c**) CD47-positive tumors contained significantly more CD163-positive TAIs (*p* < 0.0001). This is a figure. Schemes follow another format.

The clinical, pathological, and immunohistochemical features of the analyzed tumors are summarized in Table 1 and Table 2 according to CD47 and SIRPA expression, respectively. CD47 positivity showed a tendency to associate with histological differentiation (*p* = 0.032), mucus production (*p* = 0.040) and lymph node metastasis (*p* = 0.049). In contrast, SIRPA-high tumors showed a tendency to be larger (*p* = 0.037) and have mismatch-repair system deficient phenotype (*p* = 0.0072). CD47-positive tumors contained significantly higher numbers of SIRPA- (*p* = 0.044) and CD163-positive TAIs (*p* < 0.0001, Figure 3).

### 2.2. Survival Analyses of Patients with CRC

**Figure 4:**
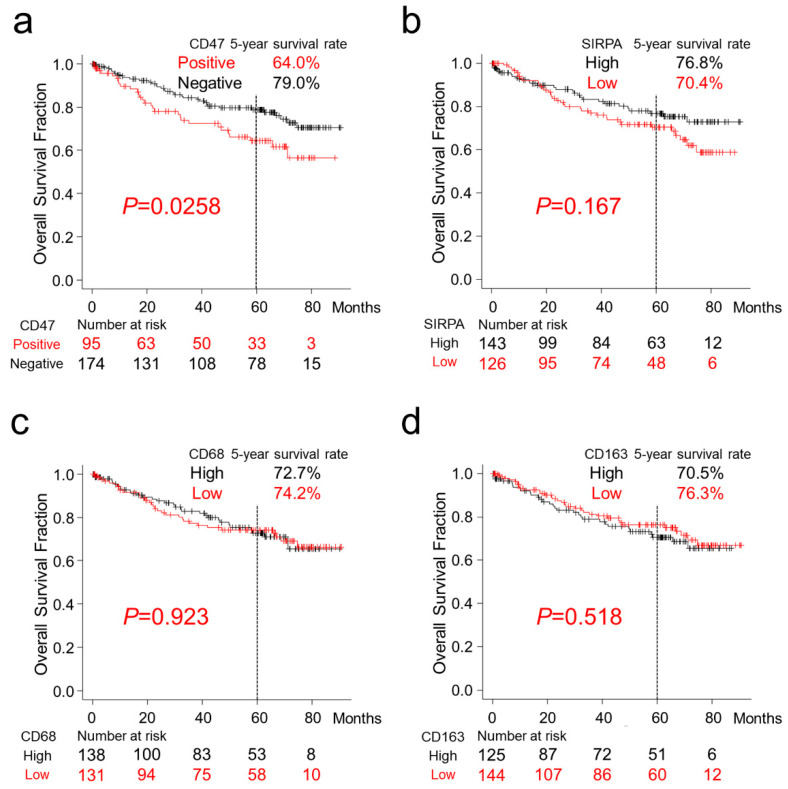
Overall survival of patients with colorectal cancer classified according to CD47, SIRPA, CD68, and CD163 expression. Kaplan–Meier curves for patients classified by CD47 (**a**), SIRPA (**b**), CD68 (**c**), and CD163 (**d**) expression. (**a**) Patients with CD47-positive CRC had significantly worse overall survival (*p* = 0.0268). (**b**–**d**) Overall survival was not correlated with the presence of SIRPA- (**b**), CD68- (**c**), or CD163-positive TAIs.

Patients with CD47-positive CRC had a significantly worse 5-year survival rate (64.0% vs. 79.0%; *p* = 0.0268). Patients with higher SIRPA-positive TAIs tended to exhibit a better 5-year survival rate (76.8% vs. 70.4%; *p* = 0.167). Overall survival was not associated with the presence of CD68- or CD163-positive TAIs (*p* = 0.923 and *p* = 0.518, respectively, Figure 4). Multivariate Cox hazards regression analysis identified tubular-forming histology (hazard ratio (HR) = 0.23, 95% confidence interval (CI) = 0.12–0.42, *p* < 0.0001), younger age (<70 years old, HR = 0.48, 95% CI = 0.27–0.83, *p* = 0.0087), and high SIRPA TAI counts (HR = 0.55, 95% CI = 0.32–0.93, *p* = 0.0027) as potential favorable factors. The analysis also revealed the presence of high tumor CD47 expression (HR = 1.75, 95% CI = 1.03–2.98, *p* = 0.038), lymph node metastasis (HR = 2.26, 95% CI = 1.31–3.91, *p* = 0.0036), and peritoneal metastasis (HR = 5.80; 95% CI = 3.23–10.43, *p* < 0.0001) as potential independent risk factors for patients with CRC (Table 3).

## 3. Discussion

Aberrant expression of CD47, a key molecule for the macrophage checkpoint, has been documented in hematological and solid malignancies with poor clinical outcomes [13,14]. Tumor CD47 expression has been suggested to contribute to immune evasion by tumor cells through the CD47–SIRPA axis. In the present study, 269 advanced CRC lesions were immunohistochemically evaluated for CD47, SIRPA, CD68, and CD163 expression in tumor cells and TAIs. Moreover, the association of their expression with clinicopathological parameters or clinical outcomes was analyzed to assess their potential for prognostication and the application of CD47–SIRPA-modulating therapy.

CD47 regulates physiological functions, including cell growth, cell migration, axon extension, cytokine production, and T-cell activation [8,9,10,11,12], as well as cancer cell proliferation, motility, and invasiveness [17,21]. Furthermore, CD47 has been reported to regulate epithelial–mesenchymal transition (EMT) and cancer stemness [22]. In the present study, CD47 expression on CRC cells was observed in 35% of lesions (95/269), and it was significantly associated with poor clinical outcomes. However, no significant association was found between CD47 expression and pT stage or histological differentiation, both of which are indicative of invasiveness and EMT phenotypes (Table 1). In addition, there was no correlation of CD47 expression and cellular proliferation (Figure S2). Furthermore, CD47-positive tumors contained significantly higher numbers of SIRPA-positive TAIs (*p* = 0.044, Figure 3a). These observations suggest that limitedly-expressed CD47-dependent activation of the CD47–SIRPA signaling in the CRC microenvironment has a significant impact on the clinical outcome of CRC, as similarly observed for other malignancies [13,14].

SIRPA is expressed on phagocytes such as monocytes, macrophages, and granulocytes, and it plays a key role in the macrophage checkpoint [15]. Evidence of the prognostic significance of SIRPA in malignancies is accumulating [23,24,25]; however, the expression and prognostic significance of SIRPA have never been reported in CRC. It is intriguing that SIRPA-high tumors showed a tendency to have mismatch-repair system deficient phenotype (*p* = 0.0072) in the present study. This might indicate the higher immunogenic potential of the mismatch-repair system deficient tumors. Differing from past reports indicating poor prognoses in patients with hematological malignancy containing SIRPA-high TAIs [23,24,25], the present study demonstrated that high SIRPA TAI counts (HR = 0.55, *p* = 0.0027) are a potential favorable factor. The discrepancies in these findings might have resulted from differences in the tumor types or the variability of TAIs. Specifically, granulocytes with SIRPA-expression are more abundant in CRC stroma (Figure S3) than those observed in hematological malignancies. A larger cohort with a longer follow-up or studies using multiple immunofluorescence staining may optimize the prognostication models.

Macrophages comprise a heterogeneous immune cell population with diverse origins and functions. Macrophages are classified into M1 and M2 macrophages. M1 macrophages are activated pro-inflammatory cells that promote inflammation and/or type 1/Th1/Th17 immune responses, whereas M2 macrophages are alternatively activated anti-inflammatory cells that prevent or antagonize inflammation and/or promote type 2/Th2 immune responses [4]. Tumor-associated macrophages (TAMs) are often found in the tumor microenvironment, and their prognostic significance has been reported in many types of cancers [26,27,28,29,30]. However, their significance regarding the survival of patients with CRC is controversial [31,32]. In the present study, no correlation was found between patient survival and the presence of CD68-positive or CD163-positive TAMs. By contrast, high SIRPA expressed in the TAIs in the tumor microenvironment was identified as a potential independent favorable factor (HR = 0.55, *p* = 0.027). These observations indicate that the subpopulations of CD68- and CD163-positive macrophages or granulocytes with SIRPA expression have uniquely important roles in modulating the clinical outcome of patients with CRC. Further investigation is needed to uncover its mechanism.

CD47–SIRPA axis-targeting agents can induce the phagocytosis of cancer cells by macrophages, which results in growth inhibition and regression of cancers in experimental conditions [14,16,17]. Recently, Hu5F9-G4, a humanized IgG4 monoclonal antibody against human CD47, was examined in phase I studies, and it displayed anti-tumor effects against both hematological and solid malignancies [18,20]. Pro-phagocytic signals such as calreticulin and phosphatidylserine on cancer cells were considered to accelerate cancer cell-specific phagocytosis [33,34]. Furthermore, the anti-tumor effects of T-cell responses was expected by the cross-presentation of tumor antigens by phagocytes to T-cells [35,36]. When Hu5F9-G4 was applied to patients with malignant lymphoma who received rituximab, an anti-CD20 antibody, Hu5F9-G4–mediated phagocytosis was augmented [13,18]. Rituximab induces complement and natural killer cell-mediated, antibody-dependent cellular cytotoxic effects via its active Fc effector function. In addition, the Fc region of rituximab provides a potent pro-phagocytic signal for macrophages by stimulating antibody-dependent cellular phagocytosis [13,18]. Based on these observations, both monotherapy with CD47–SIRPA axis-targeting drugs and combination therapy with other anti-cancer antibodies may be applied to patients with CRC.

## 4. Materials and Methods

### 4.1. Tissue Samples

The Institutional Ethical Review Board of Aichi Medical University Hospital approved this project could be performed without collecting patient consent by giving them the opportunity for opt-out. Two hundred sixty-nine formalin-fixed, paraffin-embedded (FFPE) samples of primary colorectal tumors resected at the Aichi Medical University Hospital from 2009 to 2012 were collected depending on the availability of tissue samples and clinical information. After surgery, patients were followed up for up to 90 months. All tumors were diagnosed as invasive and naïve to chemotherapy or radiotherapy. Staging of tumors was performed according to the TNM Classification of Malignant Tumors, Eighth Edition [37]. Tumors with glandular formation (>50%) or mucus production (>50% of the area) were defined to have a differentiated or mucus-producing histology. A single 4.5-mm core tumor tissue sample derived from an FFPE specimen was assembled into multitumor blocks containing up to 30 samples. All cores were obtained from invasive areas, and approximately 20% of cores contained an invasive front. The size of tumor tissue samples was estimated to exceed the size of a single 0.6-mm^2^ core by a factor of 8–9.

Non-neoplastic colonic mucosae adjacent to the tumor were also immunohistochemically analyzed.

### 4.2. Immunohistochemistry

The antibodies used in the present study are summarized in Table S1. Immunohistochemistry was performed using a Leica Bond-Max (Leica Biosystems, Bannockburn, IL, USA) or Ventana BenchMark XT automated immunostainer (Roche Diagnostics, Basel, Switzerland). In the Leica Bond-Max, antigen retrieval was performed using high pH buffer for 20 min and antibodies were applied as follows: first antibody (15 min at RT), second antibody (8 min at RT). In the Ventana BenchMark XT, antigen retrieval was performed using high pH buffer for 60 min and antibodies were applied as follows: first antibody (20 min at 37 °C), second antibody (8 min at 37 °C), Signals were visualized using 3,3′-diaminobenzidine. Following the manufacturer’s protocol, human placenta was used as a positive control for CD47 immunostaining. For SIRPA, CD68, and CD163 immunostaining, granulocytes and/or macrophages within the tissue samples were used as internal positive controls. CD47 expression on the cytomembrane of tumor cells was independently evaluated by two researchers (AS-N and ShI). The concordance rates of the initial immunohistochemical evaluation are presented in Table S2. For the discordant cases, the results were confirmed via discussion. SIRPA-, CD68-, and CD163-positive TAIs were evaluated using ImageJ software (NIH, Bethesda, MD, USA; Figure S4). Ki-67 labeling indices were determined by counting over 500 tumor cells per case in a high-power field (HPF, ×400).

### 4.3. Fluorescent Immunohistochemistry

Antigen retrieval was performed using HISTOFINE deparaffinization and antigen retrieval buffer pH 9 (Nichirei Biosciences, Tokyo, Japan) according to the manufacturer’s protocol. After blocking, primary antibodies were applied at room temperature for 1 h. The dilution of antibodies is summarized in Table S1. Signals were visualized using secondary antibodies labeled with fluorescein or tetramethylrhodamine applied at a dilution of 1:500 (Molecular Probes^®^, Thermo Fisher Scientific K. K., Tokyo, Japan). Autofluorescence was attenuated using Vector TrueVIEW Autofluorescence Quenching Kit (Vector Laboratories, INC, Burlingame, CA, USA).

### 4.4. Statistical Analyses

Statistical analyses were performed using EZR software version 1.41 [38]. The cutoffs for immunohistochemistry were defined as the value closest to the upper-left corner in the receiver operating characteristic curves for patient survival as follows: CD47, 5%; SIRPA, 226 pixels; CD68, 1743 pixels; CD163, 1027 pixels (Figure S5). The chi-squared test, Fisher’s exact test, the Cochran–Armitage trend test, Mann–Whitney’s U test, or the Kruskal–Wallis test was performed to analyze the statistical correlation between categorical data. Simple Bonferroni correction for multiple hypothesis testing was applied for adjustment at a two-sided alpha level at 0.0042 (=0.05/12).

For survival analyses, Kaplan–Meier survival estimates were calculated together with the log-rank test. Cox proportional hazards regression analysis was used to analyze the associations of survival with other factors. The initial model included variables as follows: sex (male vs. female), age (<70 years old vs. ≥70 years old), tumor size (<5 cm vs. ≥5 cm), primary tumor location (right-sided colon vs. left-sided colon vs. rectum), pT stage (pT2 vs. pT3 vs. pT4), tumor histology (moderately to well-differentiated vs. poorly differentiated), mucus production (positive vs. negative), lymph node metastasis (positive vs. negative), peritoneal metastasis (positive vs. negative), distant organ metastasis (positive vs. negative), operation status (complete vs. incomplete resection), mismatch-repair system status (deficient vs. preserved), immunohistochemical data (CD47-positive vs. CD47-negative, SIRPA-high vs. SIRPA-low, CD68-high vs. CD68-low, and CD163-high vs. CD163-low). A backward elimination with a threshold of *p* < 0.05 was used to select variables in the final model.

## 5. Conclusions

The present study demonstrated the significantly higher expression of CD47 and SIRPA in patients with CRC. Immunohistochemistry for CD47 and SIRPA could be used for the prognostication of patients with CRC. CD47–SIRPA axis-modulating therapies may be candidate treatments for patients with CRC.

## Figures and Tables

**Table 1:** Characteristics of colorectal carcinomas with or without CD47 expression. a, *p*-values were calculated by the Chi-square test for CD47 expression. b, *t*-test or c, Cochran–Armitage trend test was used to calculate *p*-values. The Bonferroni-corrected *p*-value for significance was *p* ≈ 0.0042 (0.05/12).

		Total No.		CD47 Positive		CD47 Negative		*p*-Value	
		269	(100%)	95	(35%)	174	(65%)		
Sex									a
	Male	143	(53%)	56	(59%)	87	(50%)	0.20	
	Female	126	(47%)	39	(41%)	87	(50%)		
Age, years (mean ± S.D.)		68.6 ± 12.6		67.1 ± 14.6		69.4 ± 11.3		0.15	b
Size, cm (mean ± S.D.)		5.0 ± 2.6		4.8 ± 2.4		5.1 ± 2.6		0.32	b
Tumor location									a
	Right-sided colon	124	(46%)	42	(44%)	82	(47%)	0.89	
	Left-sided colon	86	(32%)	31	(33%)	55	(32%)		
	Rectum	59	(22%)	22	(23%)	37	(21%)		
pT stage									c
	pT2	36	(13%)	17	(18%)	19	(11%)	0.50	
	pT3	189	(70%)	61	(64%)	128	(74%)		
	pT4	44	(16%)	17	(18%)	27	(15%)		
Histological differentiation									a
	Well to moderately	242	(90%)	80	(84%)	162	(93%)	0.032	
	Poorly	27	(10%)	15	(16%)	12	(7%)		
Mucus production									a
	Positive	14	(5%)	9	(9%)	5	(3%)	0.040	
	Negative	255	(95%)	86	(91%)	163	(97%)		
Lymph node metastasis									a
	Positive	124	(49%)	52	(58%)	72	(44%)	0.049	
	Negative	129	(51%)	38	(42%)	91	(56%)		
Peritoneal metastasis									a
	Positive	50	(19%)	18	(19%)	32	(18%)	1	
	Negative	219	(81%)	77	(81%)	142	(82%)		
Distant organ metastasis									a
	Positive	44	(16%)	17	(18%)	27	(16%)	0.61	
	Negative	225	(84%)	78	(82%)	147	(84%)		
Operation status									a
	Complete resection	237	(88%)	85	(89%)	152	(87%)	0.70	
	Incomplete resection	32	(12%)	10	(11%)	22	(13%)		
MMR system status									a
	Deficient	31	(12%)	14	(15%)	17	(10%)	0.24	
	Preserved	238	(88%)	81	(85%)	157	(90%)		

**Table 2:** Characteristics of colorectal carcinomas with high or low SIRPA expression. a, *p*-values were calculated by the Chi-square test for CD47 expression. b, *t*-test or c, Cochran–Armitage trend test was used to calculate *p*-values. The Bonferroni-corrected *p*-value for significance was *p* ≈ 0.0042 (0.05/12).

		Total No.		SIRPA High		SIRPA Low		*p*-Value	
		269	(100%)	143	(35%)	126	(65%)		
Sex									a
	Male	143	(53%)	73	(51%)	70	(56%)	0.50	
	Female	126	(47%)	70	(49%)	56	(44%)		
Age, years (mean ± S.D.)		68.6 ± 12.6		67.9 ± 12.6		69.4 ± 12.6		0.37	b
Size, cm (mean ± S.D.)		5.0 ± 2.6		5.3 ± 2.8		4.7 ± 2.4		0.037	b
Tumor location									a
	Right-sided colon	124	(46%)	67	(47%)	57	(45%)	0.78	
	Left-sided colon	86	(32%)	47	(33%)	39	(31%)		
	Rectum	59	(22%)	29	(20%)	30	(24%)		
pT stage									c
	pT2	36	(13%)	22	(15%)	14	(11%)	0.37	
	pT3	189	(70%)	100	(70%)	89	(71%)		
	pT4	44	(16%)	21	(15%)	23	(18%)		
Histological differentiation									a
	Well to moderately	242	(90%)	128	(90%)	114	(90%)	0.95	
	Poorly	27	(10%)	15	(10%)	12	(10%)		
Mucus production									a
	Positive	14	(5%)	4	(3%)	10	(8%)	0.11	
	Negative	255	(95%)	139	(97%)	116	(92%)		
Lymph node metastasis									a
	Positive	124	(49%)	64	(47%)	60	(51%)	0.59	
	Negative	129	(51%)	72	(53%)	57	(49%)		
Peritoneal metastasis									a
	Positive	50	(19%)	25	(17%)	25	(20%)	0.71	
	Negative	219	(81%)	118	(83%)	101	(80%)		
Distant organ metastasis									a
	Positive	44	(16%)	22	(15%)	22	(17%)	0.77	
	Negative	225	(84%)	121	(85%)	104	(83%)		
Operation status									a
	Complete resection	237	(88%)	121	(85%)	116	(92%)	0.090	
	Incomplete resection	32	(12%)	22	(15%)	10	(8%)		
MMR system status									a
	Deficient	31	(12%)	24	(17%)	7	(6%)	0.0072	
	Preserved	238	(88%)	119	(83%)	119	(94%)		

**Table 3:** Multivariable Cox hazards analysis of colorectal cancer patients. The multivariable Cox hazards analysis model initially included sex, age, tumor size, primary tumor location, pT stage, tumor histology, mucus production, lymph node metastasis, peritoneal metastasis, distant organ metastasis, operation status, mismatch-repair system status, and immunohistochemistry for CD47, SIRPA, CD68, and CD163. A backward elimination with a threshold of *p* = 0.05 was used to select variables in the final model. TAIs, tumor-associated inflammatory cells.

		95% CI		
	HR	Min	Max	*p*-Value
Well to moderately differentiated histology	0.23	0.12	0.42	<0.0001
Age (<70)	0.48	0.27	0.82	0.0087
TAIs SIRPA High	0.55	0.32	0.93	0.027
Tumor CD47 High	1.75	1.03	2.98	0.038
Lymph node metastasis	2.26	1.31	3.91	0.0036
Peritoneal metastasis	5.80	3.23	10.43	<0.0001

## Data Availability

The datasets used and/or analyzed during the present study are available from the corresponding author on reasonable request.

## Supplementary Materials

The following are available online at https://www.mdpi.com/1422-0067/22/5/2690/s1, Tables S1 and S2, Figures S1 to S5.

## Abbreviations

CRC	colorectal cancer
SIRPA	signal regulatory protein-alpha
HR	hazard ratio
CI	confidence interval
EMT	epithelial–mesenchymal transition
