# Supplementary material for: Expression and Prognostic Significance of CD47–SIRPA Macrophage Checkpoint Molecules in Colorectal Cancer
Source: Int J Mol Sci. 2021 Mar 7;22(5):2690. doi: 10.3390/ijms22052690 (PMC7975978; doi:10.3390/ijms22052690)
Supplement: Supplementary file 1 [file ijms-22-02690-s001.pdf]

Supplementary Materials: Expression and Prognostic Significance of CD47–SIRPA Macrophage Checkpoint Molecules in Colorectal Cancer

Akane Sugimura-Nagata, Akira Koshino, Satoshi Inoue, Aya Matsuo-Nagano, Masayuki Komura, Miho Riku, Hideaki Ito, Akihito Inoko, Hideki Murakami, Masahide Ebi, Naotaka Ogasawara, Toyonori Tsuzuki, Satoru Takahashi, Kunio Kasugai, Kenji Kasai, and Shingo Inaguma

Supplementary Table S1. Antibodies and conditions for immunohistochemistry. BM, Leica BondMax; IV, Ventana iView reagent; OV, Ventana OptiView reagent. Antigen retrieval was performed with heat activation in high pH buffer.

Genes	Reagent	DAB Dilution	Fluorescent Dilution	Antibodies
CD163	BM	200	100	10D6, Leica Biosystems (Bannockburn, IL)
CD47	BM	200	-	SP279, Abcam (Cambridge, UK)
CD68	BM	2 (pre-diluted)	1 (pre-diluted)	PG-M1, Nichirei Bioscience (Tokyo, Japan)
MLH1	OV	200	-	Clone G168-728, BD Biosciences, (Franklin Lakes, NJ)
MSH2	OV	200	-	Clone G219-1129, BD Biosciences, (Franklin Lakes, NJ)
MSH6	OV	400	-	Clone 44/MSH6, BD Biosciences, (Franklin Lakes, NJ)
PMS2	OV+Linker	50	-	Clone A16-4, BD Biosciences, (Franklin Lakes, NJ)
SIRPA	IV	100	100	EPR22930-163, Abcam (Cambridge, UK)

Supplementary Table S2. Concordance rate of initial immunostaining Evaluation.

**Immunohistochemistry**	Concordance Rate	k coefficient	95% Confidence Interval	
	(%)		Min	Max
CD47	82.7	0.63	0.54	0.73

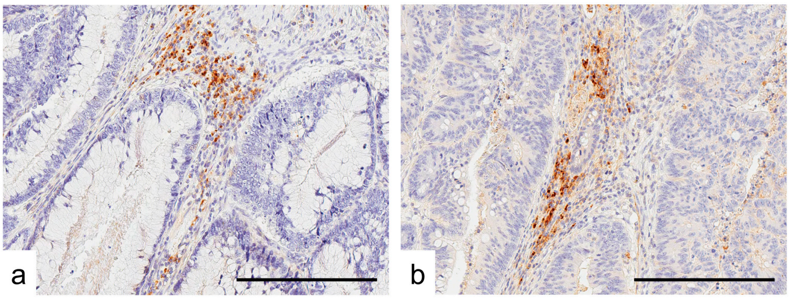

Supplementary Figure S1. Representative images for CRCs without CD47 expression. a and b, both CRC cases expressed CD47 at under-detectable levels. Note that TAIs with CD47 expression are seen. Bar, 200 µm.

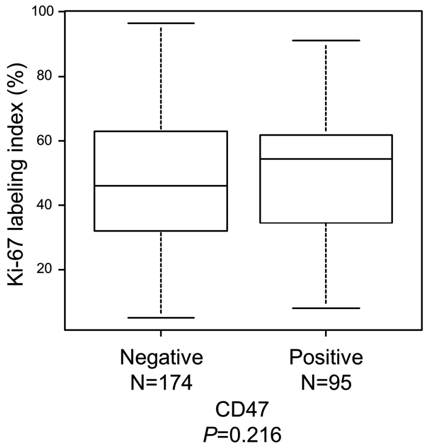

Supplementary Figure S2. The association of CD47 expression and Ki-67 labeling index in CRCs. Note that no association was detected between CD47 expression and Ki-67 labeling index (P = 0.216).

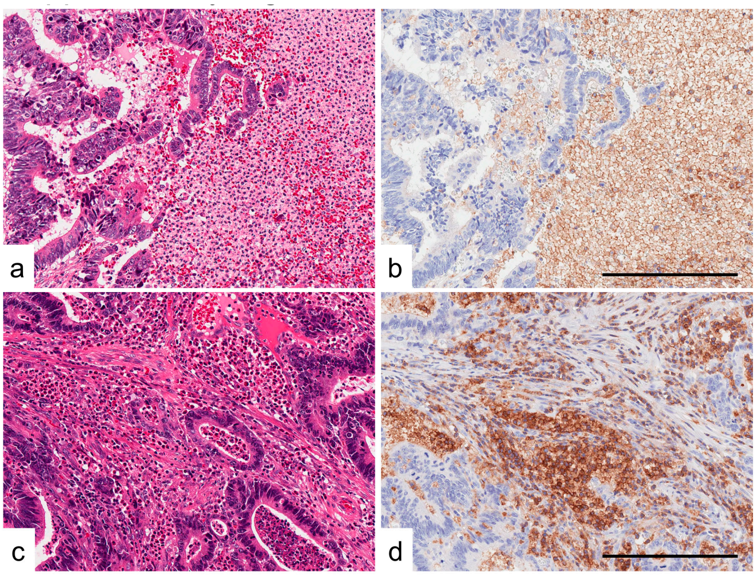

Supplementary Figure S3. Representative images for CRCs with granulocytes infiltration. (a) H&E and (b) SIRPA staining of a CRC case with neutrophile infiltration. (c) H&E and (d) SIRPA staining of a CRC case with eosinophile infiltration. Bar, 200 µm.

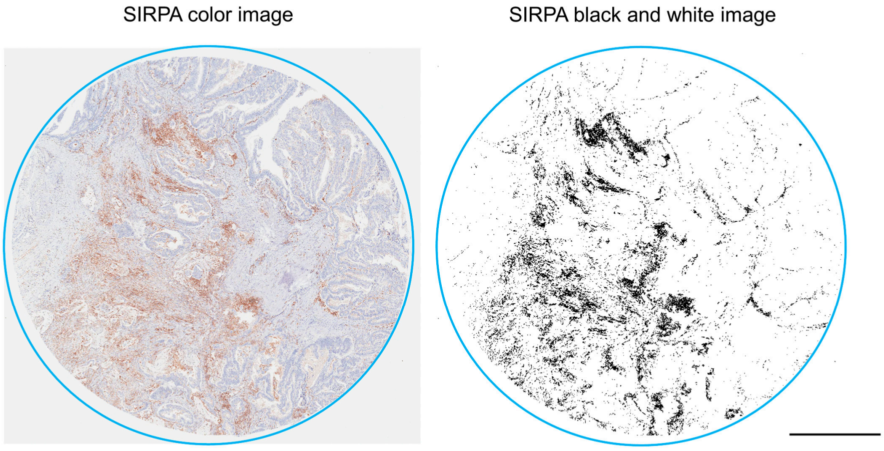

Supplementary Figure S4. Representative images for the measurement of SIRPA-positive area. Photographs for CD47 immunohistochemistry (left) were converted into black and white images (right). Black CD47-signals were measured by ImageJ software (NIH, Bethesda, MD). Bar, 1 mm.

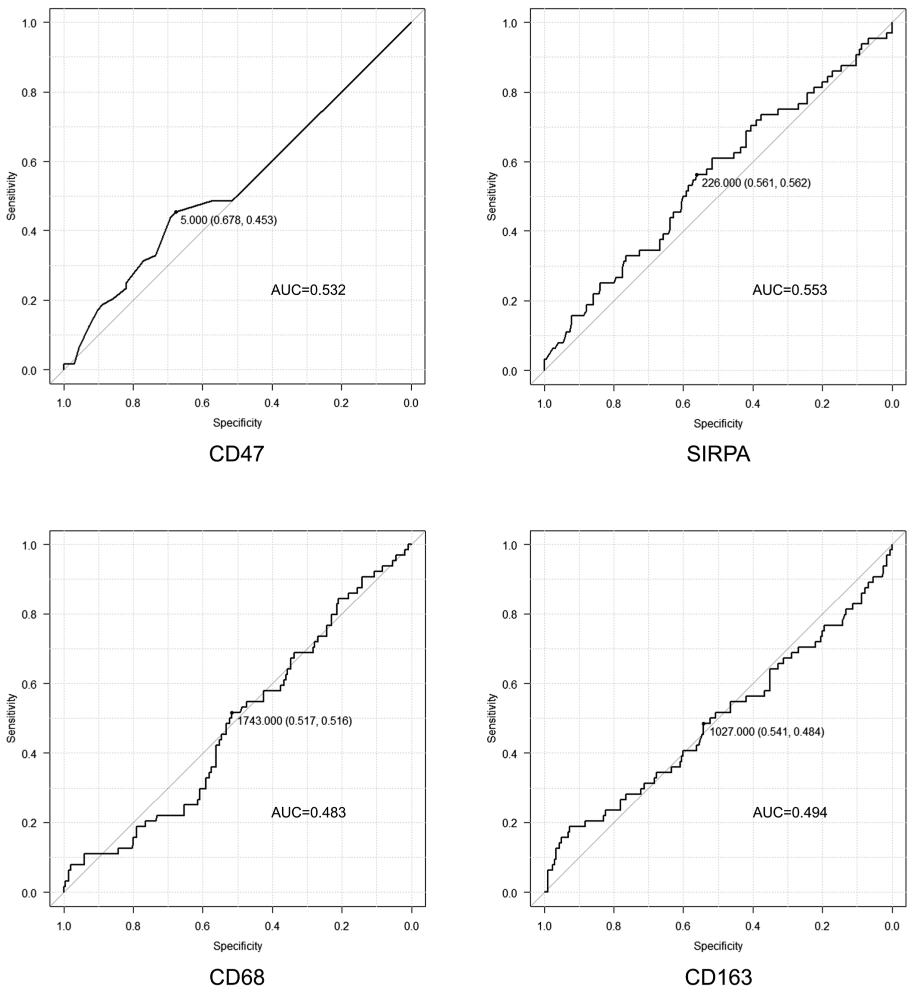

Supplementary Figure S5. ROC curves on survival of patients with CRC. ROC curves on survival of patients with CRC classified according to the expression of CD47, SIRPA, CD68, or CD163. The cut-off values for immunohistochemistry were defined using the value closest to the upper-left corner.
